# How the Sequence of a Gene Specifies Structural Symmetry in Proteins

**DOI:** 10.1371/journal.pone.0144473

**Published:** 2015-12-07

**Authors:** Xiaojuan Shen, Tongcheng Huang, Guanyu Wang, Guanglin Li

**Affiliations:** 1 Department of Information Engineering, Shaoyang University, Shaoyang, Hunan, China; 2 Key Laboratory of Human-Machine Intelligence-Synergy Systems of Chinese Academy of Sciences (CAS), Shenzhen Institutes of Advanced Technology, CAS, Shenzhen, China; 3 Department of Biology, South University of Science and Technology of China, Shenzhen, China; Swiss Institute of Bioinformatics, SWITZERLAND

## Abstract

Internal symmetry is commonly observed in the majority of fundamental protein folds. Meanwhile, sufficient evidence suggests that nascent polypeptide chains of proteins have the potential to start the co-translational folding process and this process allows mRNA to contain additional information on protein structure. In this paper, we study the relationship between gene sequences and protein structures from the viewpoint of symmetry to explore how gene sequences code for structural symmetry in proteins. We found that, for a set of two-fold symmetric proteins from left-handed beta-helix fold, intragenic symmetry always exists in their corresponding gene sequences. Meanwhile, codon usage bias and local mRNA structure might be involved in modulating translation speed for the formation of structural symmetry: a major decrease of local codon usage bias in the middle of the codon sequence can be identified as a common feature; and major or consecutive decreases in local mRNA folding energy near the boundaries of the symmetric substructures can also be observed. The results suggest that gene duplication and fusion may be an evolutionarily conserved process for this protein fold. In addition, the usage of rare codons and the formation of higher order of secondary structure near the boundaries of symmetric substructures might have coevolved as conserved mechanisms to slow down translation elongation and to facilitate effective folding of symmetric substructures. These findings provide valuable insights into our understanding of the mechanisms of translation and its evolution, as well as the design of proteins via symmetric modules.

## Introduction

Symmetry is commonly observed in the majority of soluble and membrane-bound proteins in modern cells [1]. Most of these proteins are oligomeric complexes with two or more subunits assembled in a symmetric manner [1–3]. However, some researches also demonstrated that a large number of proteins consists of monomers that have an internal symmetric structure in the monomeric state. It has been reported that six out of ten fundamental protein superfolds exhibit internal structural symmetry [4]. The role of structural symmetry in protein function and evolution, and its utility in protein engineering and design has been the focus of a number of studies [5, 6]. Structural symmetry is thought to have arisen from intragenic duplication and fusion of smaller peptide modules [7–12]: 2-fold symmetry can be generated by single gene duplication and fusion; and higher order of symmetry requires subsequent duplication and fusion. The (αβ)_8_-barrel shows 2-fold internal structural symmetry and it is suggested that (βα)_8_-barrels are derived from ‘half-barrels’ [13]. The beta-trefoil is another common protein fold that exhibits three-fold structural symmetry [14]. Notably, a recent study on membrane proteins have also revealed a strong propensity toward internal symmetric architectures [15]. Generating large symmetric proteins from small structural units using preexisting genetic material may be one of nature’s major strategies to explore the existent protein folds [16]. This evolution process may confer several significant advantages to the functions of newly innovated proteins [17, 18]. For instance, tandemly duplicated superhelical structures exhibit an extensive accessible solvent surface that is capable of binding large substrates. Symmetry may also make certain proteins more compact and thermodynamically more stable, which confers great functional advantages: these proteins become more robust to mutations, or more resistant to destabilization induced by ligand binding.

In this paper, we focus on the relationship between mRNA sequence and protein structure from the viewpoint of symmetry. Although gene duplication and fusion have been proposed to be the origin of structural symmetry, it is agreed that sequence is much more divergent than structure. A number of methods have been developed to explore the internal similarity in protein sequences [19–21], which revealed that, for most of the structurally symmetric proteins, symmetry in sequence is typically of low level or undetectable. Previous researches have shown that when physiochemical properties of different amino acids are considered, symmetry in protein sequences can be more readily detected [22–25]. Here the intragenic symmetry for structural symmetric proteins is studied directly to explore the level of gene sequence conservation. While several other methods have been developed to detect large very similar DNA repeats [26], or tandem repeats [27], few methods have aimed at identifying small and divergent intragenic repeats that are related to protein structure. Here we shall use an improved recurrence quantification analysis to study the symmetry in both the nucleotide sequence and the triplet codon sequence [22, 28].

On the other hand, the studies on the mechanisms of protein translation have become a hotspot in recent years [29–34]. It is known that the rate of translation elongation for different regions within a single mRNAs is non-uniform [35, 36]; it may depend on several factors such as codon usage of the coding sequence, higher order of local mRNA structures, codon context and adjacent codons [37, 38]. The variations of translation rate can affect the co-translational folding process and thus have significant influence on the final conformation of the protein [39, 40]. For example, Kimchi-Sarfaty *et al*. reported that for MDR1 gene, frequent-to-rare codon substitutions appear to alter the shape of transport substrate binding site and substrate specificity of the gene product P-glycoprotein [41]. Replacement of rare codons by frequent ones for genes from *Escherichia coli* or *Saccharomyces cerevisiae* led to increased translation rate but with reduced activity of the cognate proteins [35, 42]. These evidences strongly support that the degeneracy of genetic code allows the mRNA sequences to contain an additional layer of information on protein structure.

Here we explore this issue for structural symmetric proteins. Since symmetry is a rule rather than an exception of nature, there should be some conserved mechanisms that genes adopt to modulate structural symmetry in proteins. Zhang *et al*. reported that discontinuous elongation of the peptide chain and selection of slow-translating clusters that locally slow down translation might be particularly important for multidomain proteins [40]. Tuller and colleagues reported a conserved pattern that the speed of translation is slow during the first 30–50 codons and this “ramp” should be significant for translation efficiency [43]. These results suggest that translational pauses or slow translated regions might be important during protein translation to coordinate the effective co-translational protein folding. In light of these works, we shall analyze different features of mRNAs to identify conserved mechanisms in genes that allow for an efficient folding of proteins into symmetric structure.

We carry out the proposed researches by using a set of representative proteins from the left-handed beta-helix (LβH) fold, taking advantage of our previous studies on the structural symmetry of this protein fold [44]. The LβH fold is interesting since it so far has been observed very infrequently [45]. It may represent the structure of a variety of amyloid fibrils associated with prion and Huntington’s diseases [46, 47]. The polypeptide chain coils into a left-handed helical structure formed by beta-strands and separated by loop regions. This fold generally associates with transferase activity and is presented in a broad range of taxons [48]. The structures of LβH fold have been reported to be two-fold symmetric [19, 44], which means that the structure of one single helix may be composed of two symmetric subhelices. Our results reveal that for all the proteins we studied, the same degree of two-fold intragenic symmetry can be detected, providing clear evidence of gene duplication and fusion in these proteins. Moreover, we observed a major decrease of local codon usage bias in the middle region of mRNA for all the proteins we studied, which might be a conserved feature of this protein fold. In some local segments connecting symmetric substructures, we also observed major or consecutive decreases of mRNA folding energy.

## Materials and Methods

### Data acquisition

There are four superfamilies of LβH fold, each containing a number of families and species. Because codon usage bias might be different across species, we chose one protein from every species of every family to construct a set of 18 LβH proteins. Here the only one protein from superfamily Guanosine diphospho-D-mannose pyrophosphorylase was discarded from study because of the very small size in helical structure. Among the 18 proteins, 15 were from bacteria, one from plant, and two from animals. The gene sequences of these proteins were obtained from ExPASy (http://web.expasy.org/blast/) and most of the genome data used in calculating codon usage frequency (i.e., occurrences of each codon) were obtained from genbank FTP (ftp://ftp.ncbi.nih.gov/). The calculation of codon usage frequency was based on the whole genome sequence of each species. Note that the codon usage data of the three non-bacteria species were taken from a codon usage table in http://www.kazusa.or.jp/codon/. The detailed information of each protein is presented in Table 1, including the superfamily, the protein name, the species, PDB ID, the protein segment being selected and its corresponding gene segment, and the source of the species' genome data. For the selected proteins, the α helices at N terminals or C terminals were excluded from analysis. Then, sequence alignment of the proteins was carried out to explore their evolutionary relationships (Table 2). Our results show that for the same protein from different species their sequence similarity can be very high. For example, the similarity between proteins 1LXA and 1J2Z is as high as 0.46. On the other hand, proteins of the same superfamily can have very low sequence similarity. For example, the similarity between proteins 1KK6 and 1QRE is as low as 0.15. The similarity values of protein 2F9C to most of the other proteins in the set are very high, probably because of the much longer length of this protein compared with others. Proteins 1M8N and 1LOS are different isoforms of the same protein; and thus they have very high sequence similarity. The data in this paper are available upon request to the authors.

**Table 1:** Detailed information of the dataset.

Superfamily	Protein name	Species	PDB ID	Protein segments	Gene segments	Source of genome data
Trimeric LpxA-like enzymes	UDP N-acetylglucosamine acyltransferase	*Escherichia coli K12*	1LXA	1–198	1–594	Ftp
Trimeric LpxA-like enzymes	UDP N-acetylglucosamine acyltransferase	*Helicobacter pylori 26695*	1J2Z	2–193	4–579	Ftp
Trimeric LpxA-like enzymes	Galactoside acetyltransferase	*Escherichia coli K12*	1KRR	62–185	184–555	Ftp
Trimeric LpxA-like enzymes	Maltose O-acetyltransferase	*Escherichia coli K12*	1OCX	55–183	163–549	Ftp
Trimeric LpxA-like enzymes	Xenobiotic acetyltransferase	*Pseudomonas aeruginosa*	1XAT	3–166	7–498	Ftp
Trimeric LpxA-like enzymes	Xenobiotic acetyltransferase	*Enterococcus faecium*	1KK6	1–180	1–540	Ftp
Trimeric LpxA-like enzymes	N-acetylglucosamine 1-phosphate uridyltransferase GlmU	*Escherichia coli K12*	1HV9	252–438	754–1314	Ftp
Trimeric LpxA-like enzymes	N-acetylglucosamine 1-phosphate uridyltransferase GlmU	*Streptococcus pneumoniae*	1G97	252–447	754–1341	Ftp
Trimeric LpxA-like enzymes	Glucose-1-phosphate adenylyltransferase small subunit	*Solanum tuberosum*	1YP2	390–521	1168–1563	Cdtable
Trimeric LpxA-like enzymes	gamma-carbonic anhydrase	*Methanosarcina thermophila*	1QRE	4–174	10–522	Ftp
Trimeric LpxA-like enzymes	Ferripyochelin binding protein	*Pyrococcus horikoshii*	1V3W	1–144	1–432	Ftp
Trimeric LpxA-like enzymes	Putative acetyltransferase	*Bacillus cereus*	1XHD	1–144	1–426	Ftp
Trimeric LpxA-like enzymes	Serine acetyltransferase	*Haemophilus influenzae*	1SSQ	138–241	412–723	Ftp
Trimeric LpxA-like enzymes	Hypothetical protein YdcK	*Salmonella enterica*	2F9C	3–322	7–966	Ftp
Trimeric LpxA-like enzymes	Acetyltransferase PglD	*Campylobacter jejuni*	3BSW	72–195	214–588	Ftp
An insect antifreeze protein	Thermal hysteresis protein	*Choristoneura fumiferana (5-turn isoforms)*	1LOS	3–90	7–270	Cdtable
An insect antifreeze protein	Thermal hysteresis protein	*Choristoneura fumiferana(7-turn isoforms)*	1M8N	2–121	4–363	Cdtable
Adhesin YadA, collagen-binding domain	Cell adhesion	*Yersinia enterocolitica*	1P9H	32–209	94–627	Ftp

**Table 2:** Similarity (S) of the sequence alignment.

S	1LXA	1J2Z	1KRR	1OCX	1XAT	1KK6	1HV9	1G97	1YP2	1QRE	1V3W	1XHD	1SSQ	2F9C	3BSW	1LOS	1M8N	1P9H
1LXA	1	0.46	0.4	0.36	0.27	0.21	0.22	0.23	0.32	0.27	0.33	0.33	0.36	0.38	0.39	0.44	0.32	0.25
1J2Z	0.46	1	0.35	0.38	0.26	0.18	0.27	0.24	0.31	0.19	0.35	0.33	0.32	0.35	0.35	0.41	0.3	0.2
1KRR	0.4	0.35	1	0.48	0.37	0.45	0.35	0.4	0.2	0.33	0.31	0.35	0.21	0.51	0.26	0.24	0.18	0.31
1OCX	0.36	0.38	0.48	1	0.38	0.45	0.33	0.42	0.23	0.32	0.28	0.28	0.19	0.5	0.21	0.23	0.17	0.29
1XAT	0.27	0.26	0.37	0.38	1	0.4	0.27	0.26	0.22	0.2	0.24	0.25	0.18	0.43	0.3	0.31	0.24	0.22
1KK6	0.21	0.18	0.45	0.45	0.4	1	0.21	0.19	0.3	0.15	0.34	0.27	0.31	0.36	0.3	0.4	0.27	0.16
1HV9	0.22	0.27	0.35	0.33	0.27	0.21	1	0.4	0.38	0.2	0.28	0.32	0.32	0.41	0.35	0.4	0.34	0.16
1G97	0.23	0.24	0.4	0.42	0.26	0.19	0.4	1	0.38	0.21	0.33	0.32	0.36	0.41	0.4	0.43	0.32	0.18
1YP2	0.32	0.31	0.2	0.23	0.22	0.3	0.38	0.38	1	0.27	0.2	0.21	0.23	0.45	0.19	0.28	0.12	0.26
1QRE	0.27	0.19	0.33	0.32	0.2	0.15	0.2	0.21	0.27	1	0.34	0.3	0.35	0.43	0.31	0.4	0.27	0.18
1V3W	0.33	0.35	0.31	0.28	0.24	0.34	0.28	0.33	0.2	0.34	1	0.4	0.21	0.48	0.34	0.34	0.21	0.26
1XHD	0.33	0.33	0.35	0.28	0.25	0.27	0.32	0.32	0.21	0.3	0.4	1	0.31	0.49	0.19	0.32	0.21	0.23
1SSQ	0.36	0.32	0.21	0.19	0.18	0.31	0.32	0.36	0.23	0.35	0.21	0.31	1	0.46	0.27	0.3	0.12	0.32
2F9C	0.38	0.35	0.51	0.5	0.43	0.36	0.41	0.41	0.45	0.43	0.48	0.49	0.46	1	0.54	0.52	0.49	0.38
3BSW	0.39	0.35	0.26	0.21	0.3	0.3	0.35	0.4	0.19	0.31	0.34	0.19	0.27	0.54	1	0.29	0.22	0.31
1LOS	0.44	0.41	0.24	0.23	0.31	0.4	0.4	0.43	0.28	0.4	0.34	0.32	0.3	0.52	0.29	1	0.68	0.38
1M8N	0.32	0.3	0.18	0.17	0.24	0.27	0.34	0.32	0.12	0.27	0.21	0.21	0.12	0.49	0.22	0.68	1	0.3
1P9H	0.25	0.2	0.31	0.29	0.22	0.16	0.16	0.18	0.26	0.18	0.26	0.23	0.32	0.38	0.31	0.38	0.3	1

### Methods for detecting symmetry in nucleotide sequences

We analyze symmetry in nucleotide sequences by using an improved recurrence quantification analysis [22, 28]. The method works as follows. Let *S* = *x*_1_ *x*_2_ *x*_3_…*x*_*N*_ denote a nucleotide sequence of length *N*, where *x*_*i*_ represents one of the four nucleic acids. One constructs a set of all (*N-d+1*) possible segments of *d* (*d < N*) consecutive symbols:

$$\begin{array}{l} X_{1}=x_{1}x_{2}\ldots \;x_{d}{}_{,}\\ X_{2}=\;x_{2}x_{3}\ldots x_{d+1}{}_{,}\\ \ldots \ldots .\\ X_{i}=\;x_{i}x_{i+1}\ldots x_{i+d-1}{}_{,}\\ \ldots \ldots \\ X_{N-d+1}=\;x_{N-d+}{}_{1}x_{N-d+}{}_{2}\ldots x_{N}. \end{array}$$

where *i* denotes the location of the first nucleic acid (or codon) of *X*_*i*_ in the sequence. We find how many other segments are similar to each segment *X*_*i*_ and the similarities between two segments is calculated based on Hamming distance:

$$h(x_{i},x_{j})=\{\;\begin{array}{c} 1\\ 0 \end{array}\;\begin{array}{c} x_{i}=x_{j}\\ x_{i}\neq x_{j} \end{array}$$ (1)

A segment *X*_*j*_ is defined similar to *X*_*i*_ if the percentage of identical nucleic acids is larger than a threshold value *S*_0_ (Table 3).

**Table 3:** Results and parameters.

PDB id	Nt sym	*S*_0_	Cdbias sym	*C*_0_	rCAI
1LXA	2	0.35	2	0.01	1.1702
1J2Z	2	0.35	2	0.02	1.0726
1KRR	2	0.35	2	0.01	0.9798
1OCX	2	0.35	0	0.01	1.0404
1XAT	2	0.35	2	0.015	1.4127
1KK6	2	0.35	2	0.025	1.1597
1HV9	2	0.35	2	0.008	1.2223
1G97	2	0.35	2	0.01	1.1626
1YP2	2	0.30	2	0.025	1.0605
1QRE	2	0.35	2	0.005	1.0621
1V3W	2	0.35	2	0.03	1.0368
1XHD	2	0.35	2	0.03	1.1514
1SSQ	2	0.35	0	0.015	1.1794
2F9C	2	0.30	2	0.005	1.0452
3BSW	2	0.35	2	0.015	1.3315
1LOS	2	0.30	weak	0.025	1.0293
1M8N	2	0.30	weak	0.020	1.0348
1P9H	2	0.35	2	0.005	1.0474

To assess the statistical significance of the pairwise alignment, *P*-value is calculated from the distribution of similarity scores in a large number of random sequences through randomly shuffling the original sequence. The similarity of the two aligned segments is considered statistically significant when *P*-value is lower than 0.01 (i.e., the probability of obtaining an alignment with at least the same similarity by self-alignment of shuffled sequences is lower than 0.01).

### Methods for detecting symmetry in codon sequences

Because one amino acid is coded by a group of triplet codons and synonymous codons are not used in equal frequencies, we further analyze symmetry in the codon sequence. We use a revised Codon Adaption Index (rCAI) (defined in the next section) to measure the similarity between two aligned segments of codon sequences.

Let *S* = *x*_1_ *x*_2_ *x*_3_…*x*_*N*_ denote a codon sequence of length *N*, where *x*_*i*_ represents one of the 61 codons. From the sequence *S*, one constructs a set of all (*N*−*d +* 1) possible segments of *d* (*d < N*) consecutive codons:

$$\begin{array}{l} X_{1}=x_{1}x_{2}\ldots \;x_{d}{}_{,}\\ X_{2}=\;x_{2}x_{3}\ldots x_{d+1}{}_{,}\\ \ldots \ldots .\\ X_{i}=\;x_{i}x_{i+1}\ldots x_{i+d-1}{}_{,}\\ \ldots \ldots \\ X_{N-d+1}=\;x_{N-d+}{}_{1}x_{N-d+}{}_{2}\ldots x_{N}. \end{array}$$

For each segment *X*_*i*_, we find how many other segments have similar codon usage feature to it. The rCAI value of each segment is calculated. Two segments are deemed similar if the difference between their rCAI values is smaller than a threshold value *C*_0_ (Table 3).

### Revised Codon Adaption Index

CAI is defined as the geometric mean of the relative synonymous codon usage (RSCU) values corresponding to each of the codons used in that sequence, divided by the maximum possible CAI for a gene sequence of the same amino acid composition [49, 50]. It is calculated as:

$$\begin{array}{l} CAI=CAI_{obs}/CAI_{\text{max}}\\ where\\ CAI_{obs}={\left(\prod_{k=1}^{L}RSCU_{k}\right)}^{1/L}\\ {CAI}_{\text{max}}={\left(\prod_{k=1}^{L}RSCU_{k\text{max}}\right)}^{1/L} \end{array}$$ (2)

where *RSCU*_*k*_ and *RSCU*_*max*_ is the *RSCU* value for the kth codon and the maximum *RSCU* value among the synonymous codon group of kth codon, respectively. An *RSCU* value for a codon is the observed frequency of the codon divided by the frequency expected [49]:

$$RSCU_{ij}=\frac{x_{ij}}{\frac{1}{n}\sum_{j=1}^{n_{i}}x_{ij}}$$ (3)

where *x*_*ij*_ is the number of occurrences of the *j*-th codon for *i*-th amino acid, and *n*_*i*_ is the number of synonymous codons for *i*-th amino acid. For computational effectiveness, Eq (2) is computed as:

$$CAI={\left(\overset{L}{\underset{k=1}{\prod }}x_{ij}/x_{i\text{max}}\right)}^{1/L}={\left(\overset{L}{\underset{k=1}{\prod }}w_{k}\right)}^{1/L}=\text{exp}\frac{1}{L}\overset{L}{\underset{k=1}{\sum }}\text{ln}w_{k}$$ (4)

where *w*_*k*_ is the relative adaptiveness of a codon and is denoted as *x*_*ij*_ / *x*_imax_.

In this definition, the most frequently used codon from different groups of synonymous codons makes the same contribution to the result, i.e., there are up to 20 number of *w*_*k*_ whose value is 1 thus make no difference to the *CAI* value. Our purpose is to compare the similarity of codon usage feature between any two segments, the most frequently used codons from different synonymous codon group is preferred to contribute differently. For this purpose, a little revision on the definition of *CAI* is made that the maximum possible *CAI* is substituted by the expected *CAI*:

$$rCAI=CAI_{obs}/CAI_{EXP}={\left(\overset{L}{\underset{k=1}{\prod }}RSCU_{k}\right)}^{1/L}=\text{exp}\frac{1}{L}\overset{L}{\underset{k=1}{\sum }}\text{ln}RSCU_{k}$$ (5)

The revised Codon Adaption Index (rCAI) is then used as the measurement of the similarity between codon sequences.

### Local codon usage bias: the distribution of local *CAI* values over the codon sequence

Although encoding the same amino acid, synonymous codons are used with different frequencies. Synonymous codon choice can be influenced by mutation and selection sources such as Horizontal Gene Transfer [51, 52]. Selection on synonymous codons acts to increase the thermodynamic stability of DNA and RNA structures [53], as well as to assist co-translational protein folding [54]. Some codons are translationally favorable—they are selected for accurate and efficient translation in bacteria, yeast, worm, fly and even in mammals [55, 56]. Here the local codon bias is analyzed to investigate its potential correlation with structural symmetry. The distribution of local CAI values over the codon sequence is used as a measurement. In calculating the local CAI values, the length of the sliding window is set to be 20 consecutive codons, which is chosen based on the structure feature of LβH that the unit of one rung is 18 to 20 amino acids long [57]. The sliding window moves from the beginning to the end of the codon sequence, with one codon for each move. Then the local *CAI* of all *d* = 20 sliding windows are calculated and shown as the distribution over the codon sequence.

### Local mRNA folding energy

Single-stranded mRNA sequences may form local secondary structures which is believed to have influences on protein translation. Secondary structure in the coding regions might generally be used to regulate the translation rate which might cause temporarily translation pause to facilitate protein folding. Here we study the local RNA structures alongside a codon sequence. Because the footprint length of a ribosome on mRNA is about 40 nucleotides, we calculate the local folding energy with a sliding window of 40 nucleotides downstream. The sliding window moves from the beginning to the end of the nucleotide sequence, with one nucleotide for each move. The folding energy of the segment framed by the sliding window is calculated by using Matlab function *rnafold* which predicts the minimun free energy of the RNA sequence using the thermodynamic nearest-neighbor approach (http://www.mathworks.com/help/bioinfo/ref/rnafold.html).

### Randomization

To demonstrate that the features of the local codon usage and mRNA folding energy of LβH are specially selected by nature, we perform randomization on the gene sequences for comparison. For each native gene sequence, we randomly shuffle the set of codons among sites with identical amino acids, preserving the exact count of each codon and the precise order of encoded amino acids as in the native sequence [58]. We performe 10 times of the random procedure for each of the gene sequence and calculate the average profiles of both local codon usage bias and folding free energy. The averaged profiles for 10 random sequences are then compared with that of the natural gene sequences.

## Results and Discussion

### The gene sequence of protein 1LXA is two-fold symmetric

**Fig 1:**
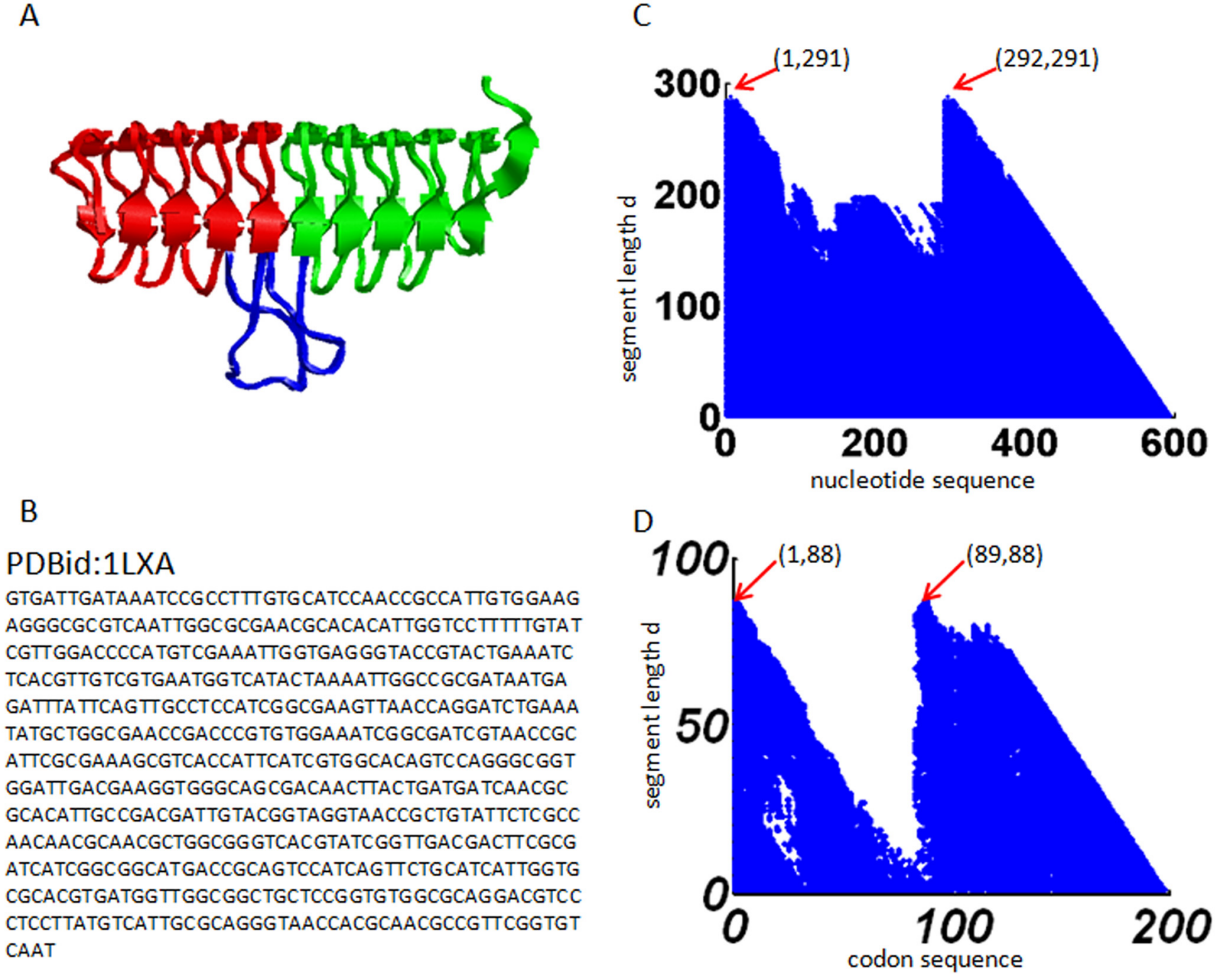
Results obtained from protein 1LXA. (A) The gene sequence. (B) The tertiary protein structure. (C) The recurrence plot of the nucleotide sequence. (D) The recurrence plot of the codon sequence.

The protein 1LXA has a two-fold symmetric tertiary structure (Fig 1A): two symmetric substructures (shown in red and green, respectively) connected by an irregular extended loop in the middle (shown in blue). We apply the method described in Section 2.2 to analyze the nucleotide sequence of this protein, which has a length *N* = 594 (Fig 1B). Here we set *S*_0_ = 0.35. The result for the nucleotide sequence (Fig 1C) shows two peaks at positions (1,291) and (292,291) which means that segments 1–291 and 292–582 are similar to each other; and thus the nucleotide sequence is two-fold symmetric. The correspondence between the symmetry in gene sequence and the symmetry in protein structure provide clear evidence of gene duplication and fusion event for this protein.

We also verify the two-fold symmetry of protein 1LXA from the perspective of codon usage bias. We apply the method described in Section 2.3 to analyze the codon sequence of this protein, which has a length *N* = 198. Result in codon usage bias (Fig 1D) also reveals a two-fold symmetry in the codon sequence, with the first segment containing codons 1–88 and the second segment containing codons 89–176. The codon sequence is thus two-fold symmetric. It suggests that the two symmetric subsegments of the gene sequence also share similar codon usage bias. Here the *C*_0_ is set to be 0.01.

### Two-fold symmetry is an evolutionarily conserved feature of LβH proteins

**Fig 2:**
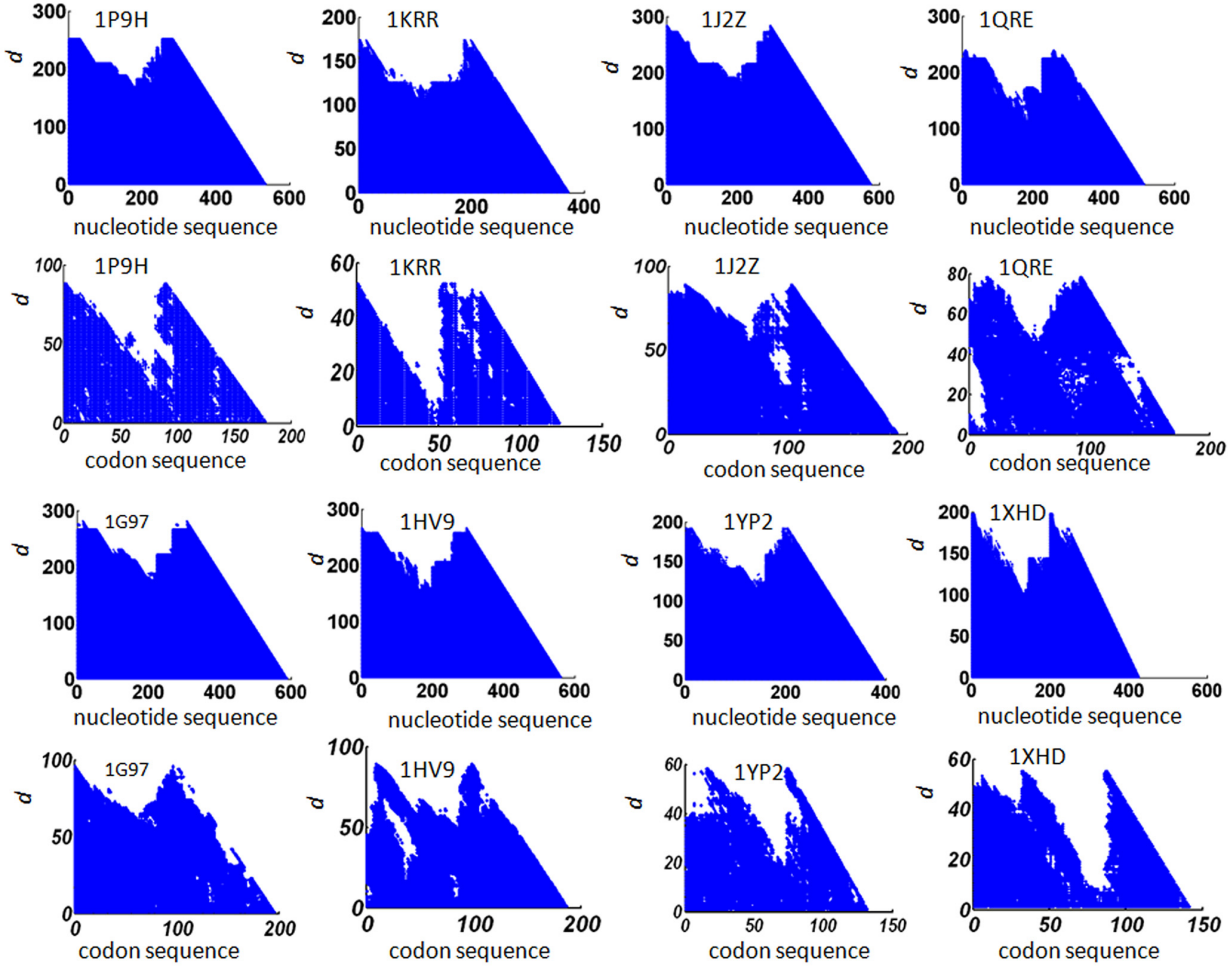
Recurrence plots for 8 proteins. The first and third horizontal panel: the recurrence plot of the nucleotide sequence; the second and the fourth horizontal panel: the recurrence plot for codon usage bias in the codon sequence of the corresponding proteins. The PDB id of the protein is given in each of the plot.

We apply the method described in Section 2.2 to analyze the nucleotide sequences of all the 18 LβH proteins listed in Table 1. The results of eight of them are presented in Fig 2; and the other results are presented in S1 Fig. For all the cases, two-fold symmetry is detected in the nucleotide sequence. The threshold for determining similarity *S*_0_ was set in the range [0.30, 0.35], which is relatively high when compared with 0.25, the similarity for two protein sequences to have similar tertiary structures [59, 60]. This result suggests that one single gene duplication and fusion event to form a two-fold symmetric tertiary structure might be a common evolution process for this protein fold.

We apply the method described in Section 2.3 to analyze the codon sequences of the 18 LβH proteins. The results of eight of them are presented in Fig 2. For 14 of the 18 cases, two-fold symmetry is detected, indicating that codon usage bias might be conserved during evolution. The threshold for determining similarity *C*_0_ was set in the range [0.005, 0.03]. The wider range of similarity degree for codon usage bias may due to the divergence of individual *revised CAI* value for each of the gene sequence. To verify the effectiveness of our recurrence analysis, we compare it with the web server Swelf [61]. The software uses dynamic programming to identify internal repeats in DNA sequences, amino acid sequences, and three dimensional structures. It turns out that Swelf could not find repeats for most of the gene sequences in our dataset.

Table 3 summarizes the results and parameters in this section, where the column“Nt sym”represents symmetry in nucleotide sequence. Because all the nucleotide sequences show two-fold symmetry, the column is labeled with 2 for all the rows. The column“Cdbias sym”represents symmetry in codon usage bias. Of the 18 sequences, 14 show two-fold symmetry, two show no symmetry, and two show weak symmetry. The column “rCAI”represents the rCAI value of the codon sequence.

### Profiles of local codon usage bias and local mRNA folding free energy

**Fig 3:**
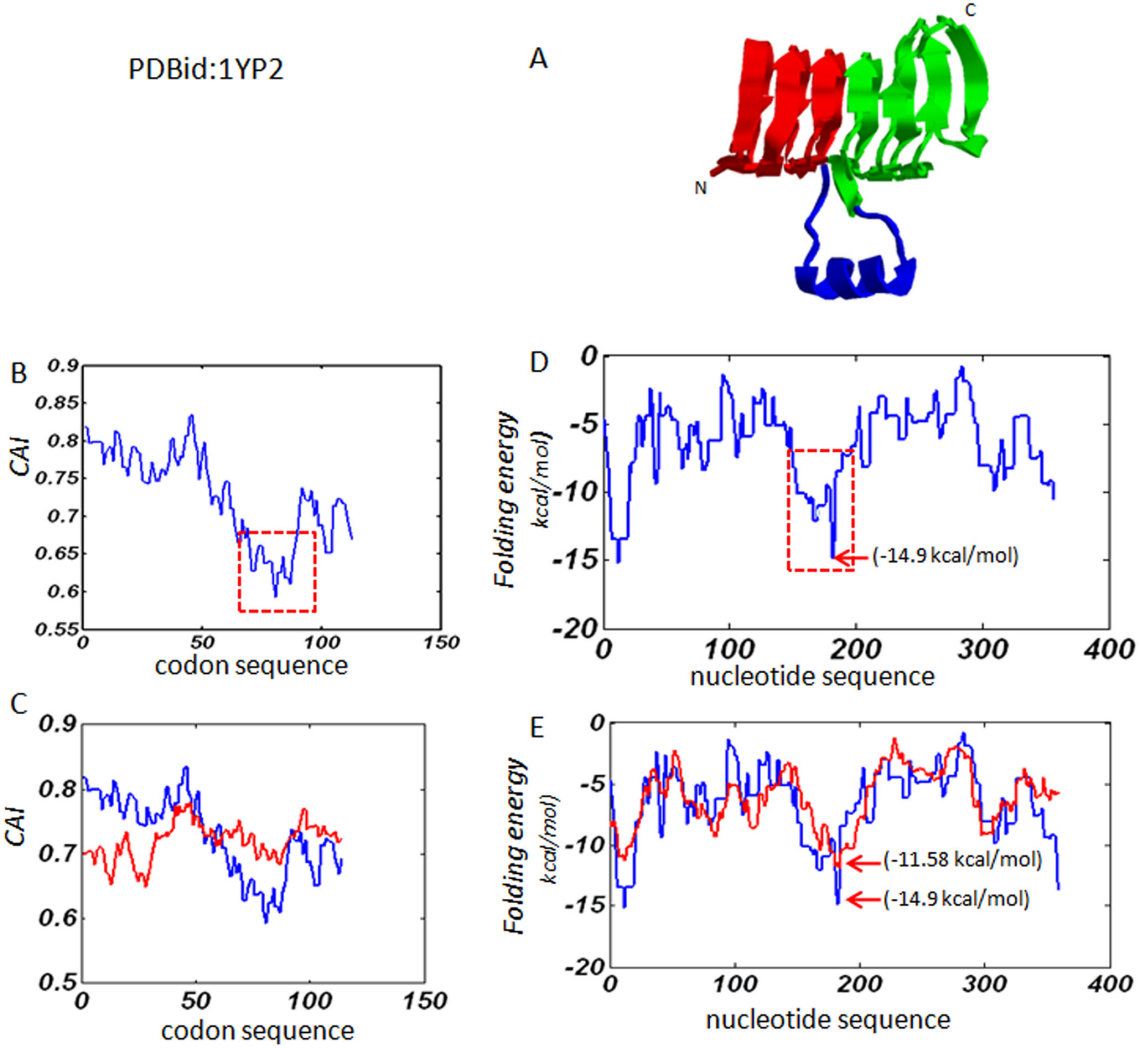
Results obtained from protein 1YP2. (A). The cartoon structure: the two symmetric substructures are shown in red and green; the extended irregular structure in the middle of the helix is shown in blue. (B). The profile of local codon usage bias: the decreased region in the middle of the codon sequence is shown with dashed square lines. (C). The comparison between the profile of the natural gene sequence and the averaged profile of the same codon sequence randomized by 10 times. The blue line is for natural gene sequence and the red line is for the average of the random sequences. (D). The profile of local folding free energy, and the dashed square lines shows the region with decreased local folding free energy. (F). The comparison between the natural gene sequence and the random sequences. The blue line is for natural gene sequence and the red line is for the average of the random sequences.

The protein 1YP2 has a two-fold symmetric tertiary structure (Fig 3A): six complete rungs (shown in red and green) connected by irregular extended structure in the middle (shown in blue). Fig 3B shows the profile of CAI values calculated along the sequence with a sliding window of length *d* = 20 codons. In the figure, a smaller CAI value indicates rarer codons are used nearby. The result suggests that rare or less frequently used codons are more likely to be used near the boundary between the two symmetric substructures. In Fig 3C, the CAI profile of 1YP2 (blue) is compared with the CAI profile (red) averaged from 10 random codon sequences of the same length. Clearly, the random profile is flat and lacks the nadir marking the boundary between two symmetric substructures. This demonstrates that the increased usage of rare codons near the connecting region is not random, but is specially selected by nature during evolution.

Fig 3D illustrates the profile of local mRNA folding energy calculated along the sequence with a sliding window of length *d* = 40 nucleotides. In the figure, a lower energy implies the mRNA folding is locally more stable. The profile also shows a major decrease of local folding free energy in the middle of the nucleotide sequence, with the lowest energy of -14.9 kcal/mol, suggesting that higher order of secondary structure might be formed near the connecting region of symmetric substructures. In Fig 3E, the folding energy profile of 1YP2 (blue) is compared with a folding energy profile (red) averaged from 10 randomly generated sequences. Although the folding energy difference between the blue curve and the red curve is less dramatic than the CAI difference, the nadir of the major decreased region of the blue curve is significantly lower than that of the red curve: -14.9 kcal/mol versus -11.58 kcal/mol (Fig 3E). These results suggest that codons near the connecting region might be selected rather than randomly chosen for the formation of more stable structure.

**Fig 4:**
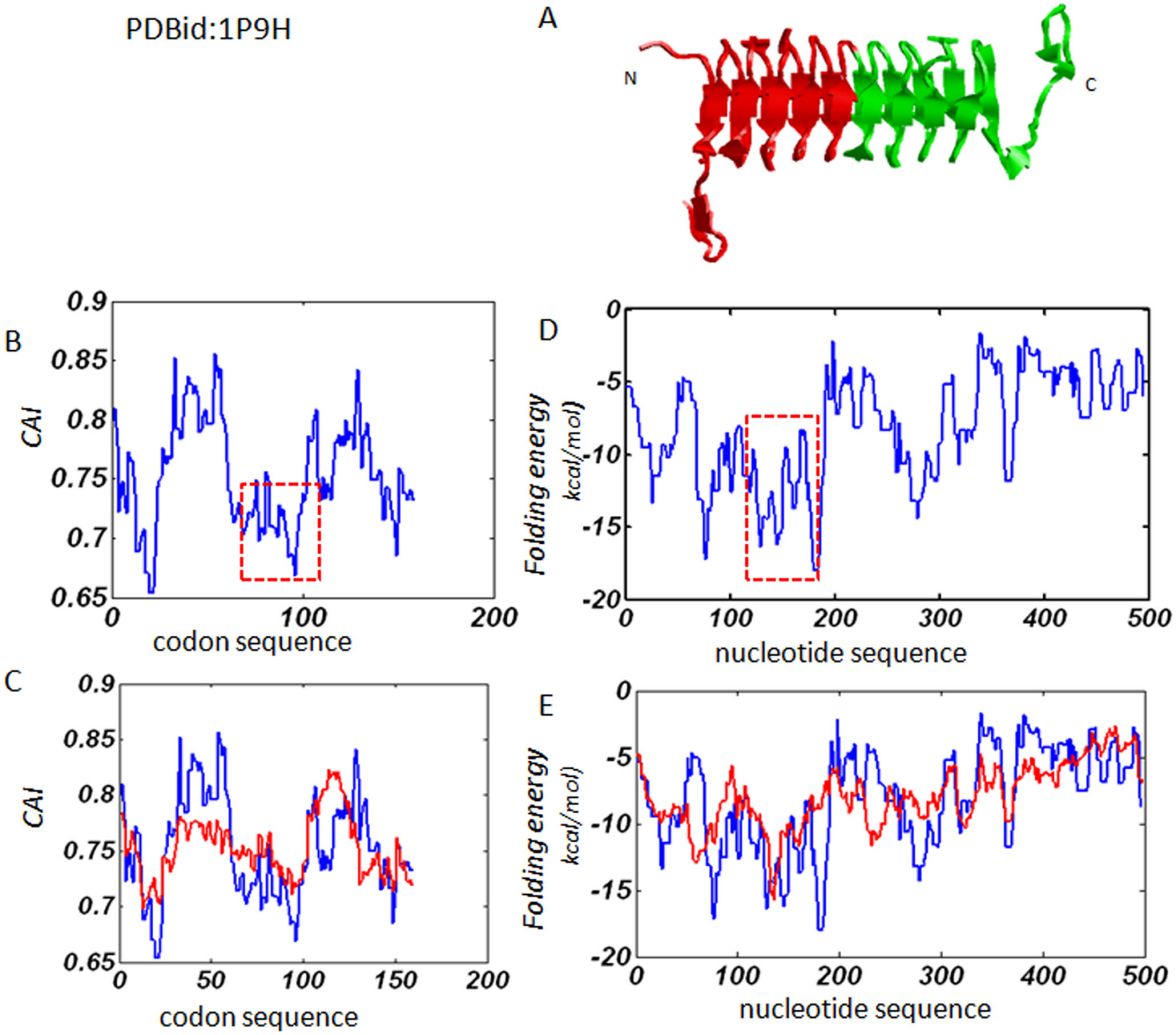
Results obtained from protein 1P9H. (A). The cartoon structure, with the two symmetric substructures shown in red and green respectively. (B). The profile of local codon usage bias: the region with major decreases in *CAI* value is shown with dashed square lines. (C). The comparison between the profile of the natural gene sequence and the averaged profile of the same codon sequence randomized by 10 times. The blue line is for natural gene sequence and the red line is for the average of the random sequences. (D) and (E). The profile of local folding free energy and the comparison between the natural gene sequence and the random sequences, respectively. The blue line is for natural gene sequence and the red line is for the average of the random sequences.

The protein 1P9H serves as another example. Unlike the structure of 1YP2, there is no extended irregular loop in the middle of the helix structure (Fig 4A). It yields results similar to 1YP2 in terms of both local codon usage bias and local mRNA folding free energy. Fig 4B shows a major decrease of local codon usage in the middle of the codon sequence. The comparison between the natural gene sequence and the average of 10 random codon sequences shows that the natural gene sequence has more biased codon usage than random sequences (Fig 4C). As such, a major decrease of local folding free energy in the middle of the nucleotide sequence is also detected (Fig 4D). Moreover, the natural gene sequence has more biased folding free energy distribution than random sequences (Fig 4E), indicating that the connecting region is more stable than the counterpart of a random sequence. Therefore, higher order of secondary structure is likely to form in the middle region.

**Fig 5:**
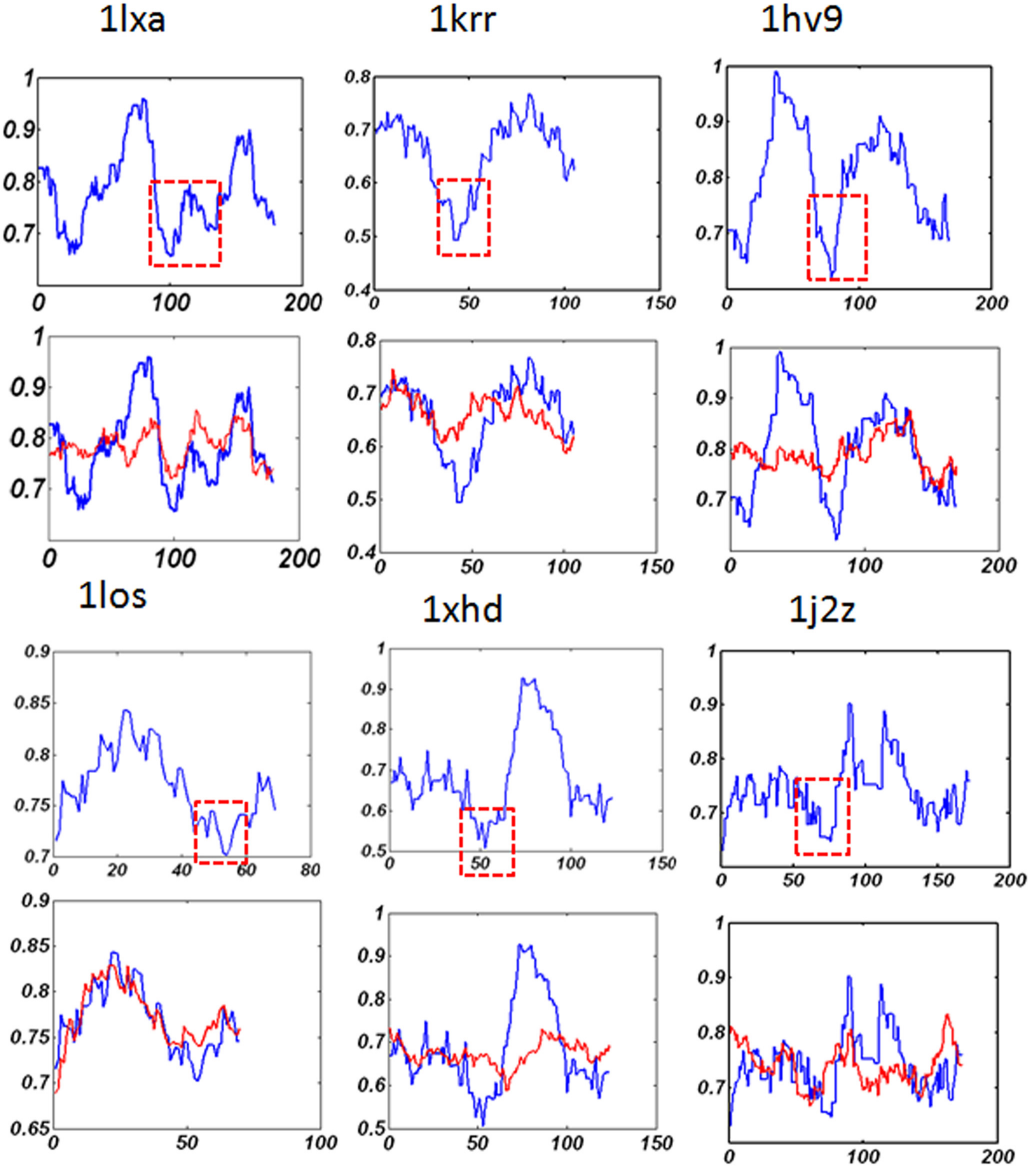
The profile for local codon usage bias and the comparison of natural gene sequences to the random sequences. The x-axis is the codon sequence and the y-axis is the local *CAI* value. The major decrease of local codon usage bias is shown with dashed square lines. The blue line is for natural gene sequence and the red line is for the average of the random sequences.

According to the tertiary structure, some LβH proteins such as 1LXA and 1YP2 contain extended loop regions in the middle of the helix structure; while some proteins such as protein 1P9H and protein 1HV9 (whose tertiary structure can be obtained from protein data bank) do not contain irregular loop region. In order to further verify that the above discovery involves a conserved feature of the entire LβH protein folds, we extend the study to all the representative sets of LβH proteins. Fig 5 gives the results for 6 LβH proteins and S2 Fig gives the results for the other 10 proteins.

**Fig 6:**
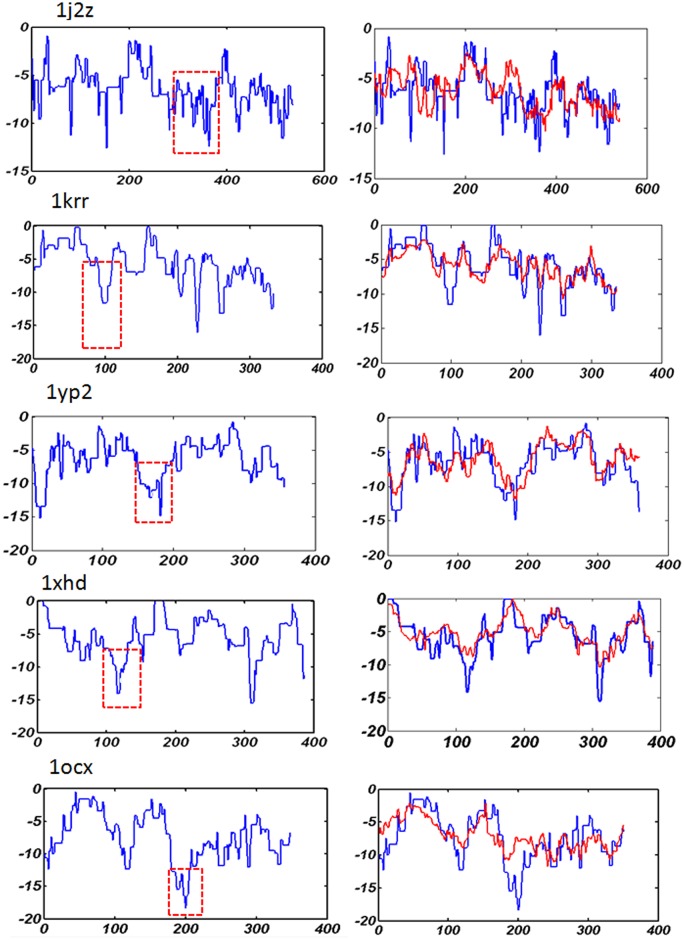
The profile for local mRNA folding free energy and the comparison of natural gene sequences to the random sequences. The x-axis is the nucleotide sequence and the y-axis is the local folding free energy. The region with major decrease of local mRNA folding free energy is shown with dashed square lines. The blue line is for natural gene sequence and the red line is for the average of the random sequences.

The study reveals that, for nearly all the representative proteins in the dataset, there is a major decrease of local codon usage bias in the middle of the codon sequence. It implies that the usage of less frequently used codons near the boundaries of the two symmetric substructures of LβH fold might be a conserved mechanism during the evolution process. Previous studies reported that the location of rare codon clusters often indicates domain termini and/or boundaries in multidomain proteins [35], and it may temporally separate the protein synthesis for hierarchical cotranslational folding of multidomains [40]. Our results on local codon usage bias suggest that these findings might also be true for single domain protein with internal structural symmetry. The study on the local folding free energy also shows that there are major or consecutive decreases of local folding free energy near the connecting region of symmetric substructures (Fig 6, and S3 and S4 Figs). Comparisons with random sequences show that natural gene sequences have more biased local folding free energy than random gene sequences. A recent study on mRNA folding suggested that mRNA secondary structures serve as elongation brakes to control the speed and hence the fidelity of protein translation [62]. Here the results in our study indicate that the tuning of translation elongation by local mRNA stability might relate to structural symmetry in proteins. Since both clusters of rare codons and formation of higher order of local mRNA secondary structure may lead to translation pause or slow-down regions, our results may suggest a sequential folding pathway of nascent peptide chain of symmetric structures.

## Conclusion

In this paper, we studied the relationship between the structural symmetry of proteins and the nucleotide sequence, codon bias, and mRNA folding energy in LβH fold. The results show that a similar degree of symmetry can be readily detected in both the nucleotide sequence and the triplet codon sequence. In addition, a major decrease of local codon usage bias in the middle of the codon sequence can be identified as a common feature. Major or consecutive decreases in local mRNA folding energy near the boundaries of the symmetric substructures can also be observed. Taken together, our results suggest that gene duplication and fusion event to form two-fold symmetric tertiary structure may be a common evolution process of LβH fold. Meanwhile, the selection over local codon usage and local mRNA secondary structure near the connecting regions of symmetric substructures might have coevolved and become a conserved mechanism for tuning translation of this protein fold. These findings provide valuable insights into the evolution and mechanisms of translation, and may also shed light on the design and engineering of symmetric protein folds.

## Supporting Information

S1 Fig The recurrence plots for 9 proteins. The first, third and fifth horizontal panel: the recurrence plot of the nucleotide sequence; the second, fourth and sixth horizontal panel: the recurrence plot for codon usage bias in the codon sequence of the corresponding proteins. The PDB id of the proteins are given in each of the plot.
(RAR)

S2 Fig The profile for local codon usage bias and the comparison of natural gene sequences to the random sequences. The x-axis is the codon sequence and the y-axis is the local CAI value. The major decrease of local codon usage bias is shown with dashed square lines. The blue line is for natural gene sequence and the red line is for the average of the random sequences.
(RAR)

S3 Fig The profile for local mRNA folding free energy and the comparison of natural gene sequences to the random sequences. The x-axis is the nucleotide sequence and the y-axis is the local folding free energy. The region with major decrease of local mRNA folding free energy is shown with dashed square lines. The blue line is for natural gene sequence and the red line is for the average of the random sequences.
(RAR)

S4 Fig The profile for local mRNA folding free energy and the comparison of natural gene sequences to the random sequences. The x-axis is the nucleotide sequence and the y-axis is the local folding free energy. The region with major decrease of local mRNA folding free energy is shown with dashed square lines. The blue line is for natural gene sequence and the red line is for the average of the random sequences.
(RAR)
